# Cell-TypeAnalyzer: A flexible Fiji/ImageJ plugin to classify cells according to user-defined criteria

**DOI:** 10.1017/S2633903X22000058

**Published:** 2022-05-20

**Authors:** Ana Cayuela López, José A. Gómez-Pedrero, Ana M. O. Blanco, Carlos Oscar S. Sorzano

**Affiliations:** 1 Biocomputing Unit, National Centre for Biotechnology, Madrid, Spain; 2 Applied Optics Complutense Group, Faculty of Optics and Optometry, University Complutense of Madrid, Madrid, Spain; 3 Advanced Light Microscopy Unit, National Centre for Biotechnology, Madrid, Spain

**Keywords:** Cell classification, cell type, fluorescence microscopy, marker, segmentation

## Abstract

Fluorescence microscopy techniques have experienced a substantial increase in the visualization and analysis of many biological processes in life science. We describe a semiautomated and versatile tool called Cell-TypeAnalyzer to avoid the time-consuming and biased manual classification of cells according to cell types. It consists of an open-source plugin for Fiji or ImageJ to detect and classify cells in 2D images. Our workflow consists of (a) image preprocessing actions, data spatial calibration, and region of interest for analysis; (b) segmentation to isolate cells from background (optionally including user-defined preprocessing steps helping the identification of cells); (c) extraction of features from each cell; (d) filters to select relevant cells; (e) definition of specific criteria to be included in the different cell types; (f) cell classification; and (g) flexible analysis of the results. Our software provides a modular and flexible strategy to perform cell classification through a wizard-like graphical user interface in which the user is intuitively guided through each step of the analysis. This procedure may be applied in batch mode to multiple microscopy files. Once the analysis is set up, it can be automatically and efficiently performed on many images. The plugin does not require any programming skill and can analyze cells in many different acquisition setups.

## Impact Statement

Cell-type classification is an absolute requirement for quantitative analysis of microscopy imaging. Because different cell types normally differ in function and appearance, this tool allows researchers to correctly identify specific cells sharing common shape, life cycle, and phenotypical features. Here, we present Cell-TypeAnalyzer, a new plugin freely available under ImageJ or Fiji distribution for automated cell detection, identification, characterization, counting, and further cell-type classification based on user-defined criteria. Our tool aims at reducing the amount of subjectivity and human labor required to quantitatively assess the outcome of an imaging experiment.

## 1. Introduction

Nowadays, both multi-fluorescence imaging and labeling techniques are commonly used to identify biologically relevant processes through quantitative data extraction from fluorescently labeled molecules of interest^(^^1^ ^–^^3^ ^)^. Parallel to this unprecedented progress, advances in open-source bio-image software and scientific computing^(^^4^ ^)^, cell counting automation, and single-particle analysis algorithms ensure reproducibility and objectivity compared to the more subjective manual analyses^(^^5^ ^,^^6^ ^)^. In cell biology, distinguishing specific cell types has traditionally been a labor-intensive and subjective task since it tries to classify cells according to morphological or phenotype forms^(^^7^ ^)^ using tedious laboratory procedures as visual inspection. It is quite challenging to design a versatile algorithm to automatically identify different cell types on multiple fluorescent markers located on the same field^(^^8^ ^,^^9^ ^)^ at the single-cell level. Additionally, in fluorescence microscopy, the signal-to-noise ratio is often low and the resolution quite limited^(^^10^ ^)^, making automation of cell-type classification even more challenging^(^^11^ ^)^. In this context, single-cell features extraction arises helping researchers to properly overcome these drawbacks defining cell types which will be then cataloged into groups revealing different cell states or behaviors.

**Figure 1.**
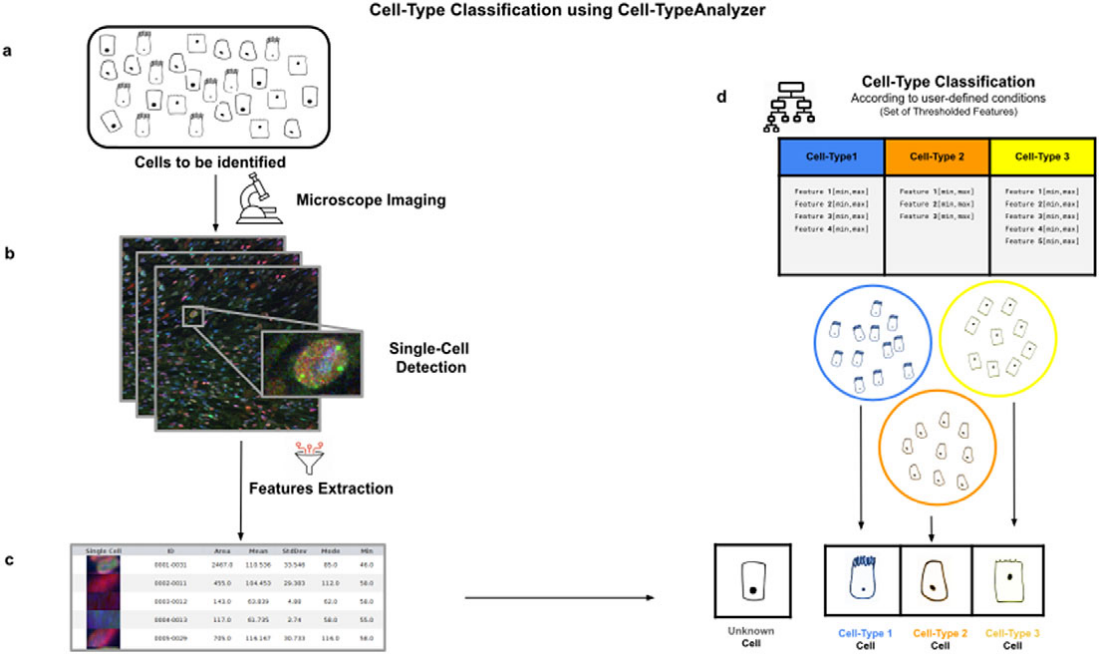
Illustration of the workflow to identify specific cell types in a cell population. (a) Cell culture in which classification will be done to identify specific cell types. (b) Cell images are acquired and then processed for single-cell segmentation, feature extraction, and cell-type classification. (c) A collection of diverse features are extracted to both characterize and identify by ID number each cell. (d) Cell types are defined by a set of constraints in any of the detected features. The user may define as many cell types as needed, and each cell type is defined by as many constraints on the features as desired.

Cell-TypeAnalyzer allows the user to classify cells of interest (see Figure 1), identifying a set of cells sharing common morphological, intensity, or spatial features according to a given biologically defined class. Cell-TypeAnalyzer is an open-source plugin under the GNU public license that works equally well under Fiji^(^^12^ ^)^ or ImageJ^(^^13^ ^)^, offering a semiautomated cell-type classification using separated RGB channels for multiple microscopy image formats in an objective manner considerably more accurate than qualitative strategies. Therefore, Cell-TypeAnalyzer enables users describing a cell population through a set of extracted features to identify biologically relevant similarities or variations on a sample.

Our tool is highly configurable and may be adapted to many density or low-resolution situations by tuning the workflow internal parameters. We do not make any special assumptions about cell morphology, image formation process, optical microscope settings, or specimen features. In quantitative immunohistochemistry analysis, holding an accurate segmentation method to exactly isolate each cell from its possible fluctuating background is crucial to reach a robust detection^(^^14^ ^,^^15^ ^)^. In particular, in cases dealing with heterogeneous staining or overlapping cells, global auto-threshold methods may be a generic option to find the global optimal segmentation^(^^16^ ^)^ grouping image pixels automatically depending on pixel values with no presumption about binary shapes or circularity, and hence leading to a less-biased detection non-exclusively limited to spot-like or roughly spherical objects^(^^11^ ^)^. Once the segmentation of each cell is done, each one is measured as well as described individually by a vector of physical, morphological, statistical, and intensity features and then used for further cell-type classification.

Apart from that, this plugin was implemented under the ImageJ ecosystem to benefit from this bio-image platform mainly preferred and used by many life scientists. In recent years, researchers may choose from a wide range of open-source bio-image packages^(^^17^ ^)^ to customize their own image analysis protocols through scripts, workflows, or plugin development. Nevertheless, many researchers may not have this computational proficiency. For such cases, Cell-TypeAnalyzer allows semi-automation based on a broadly applicable strategy for customized cell classification^(^^18^ ^)^. Furthermore, Cell-TypeAnalyzer is easily scriptable to customize the cell-type approach even in batch mode and obtain user-defined cell-type classifications across RGB channels dealing with multiple image formats currently supported by Bio-Formats^(^^19^ ^)^. Additionally, the user may choose a specific region of interest rather than considering the whole image. The researcher is guided through a user-friendly wizard-like graphical user interface (GUI) to perform each step. This GUI allows navigating forward or backward across panels to recalibrate settings in case of inadequate outputs.

## 2. Results

### 2.1. Overview of the procedure

Cell-TypeAnalyzer can work with images with up to three color channels. One of the channels, called Marker I, defines what a cell is and what is not. This channel can be a marker of cytoplasm, nuclei, or any other cellular structure of interest. Once we have identified cells with Marker I, cell types will be defined with Markers II and III.

**Figure 2.**
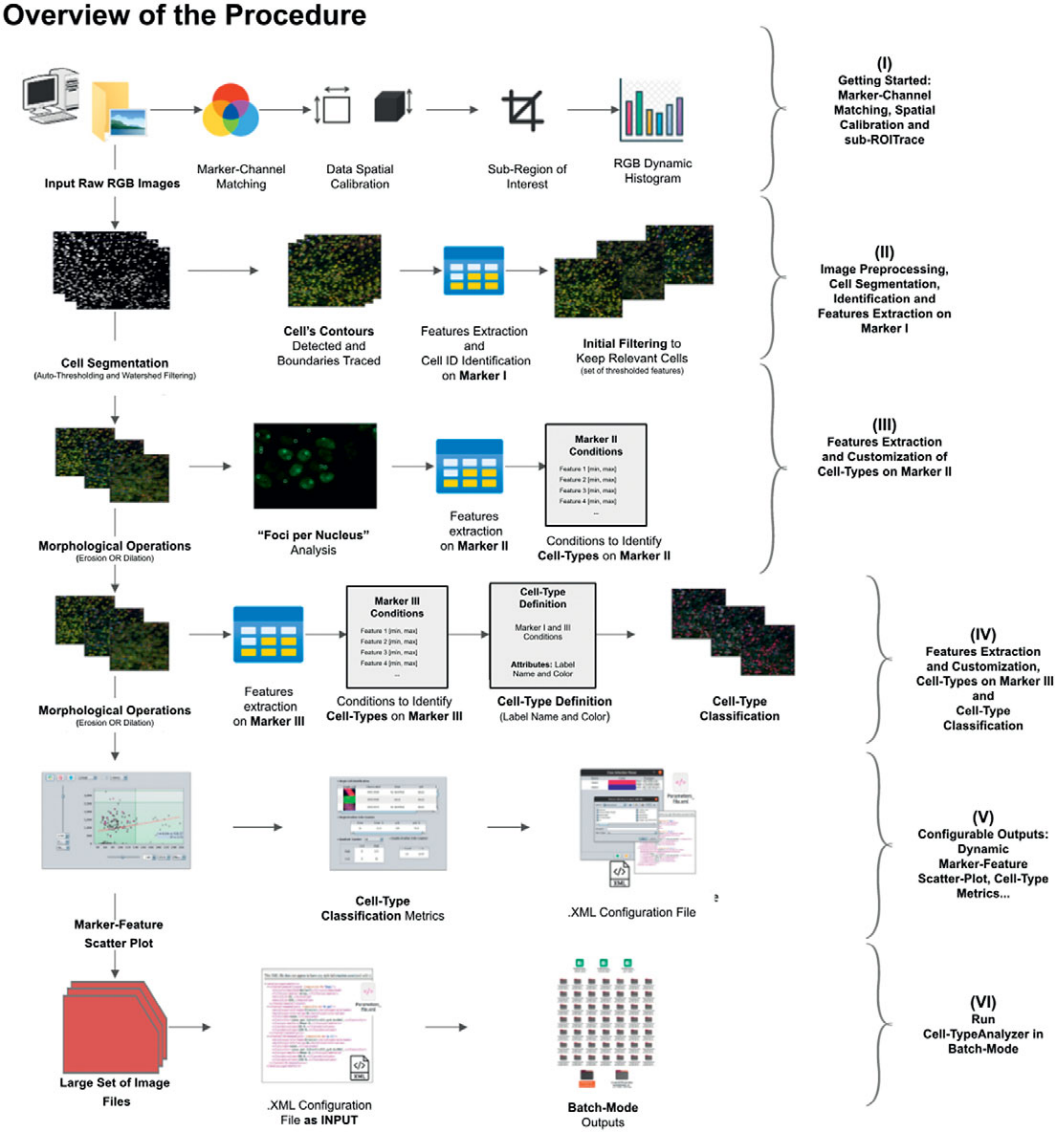
Schematic overview of the Cell-TypeAnalyzer procedure to classify cells. (I) Marker-Channel Matching, data spatial calibration to have measurements on physical units, drawing a region of interest to restrict cell classification to a specific area. (II) Image preprocessing actions, cell segmentation (auto-thresholding and watershed transformation) to isolate cells from their background, identification by ID number, and feature extraction on Marker I. (III) Cell features are extracted on Marker II and declaration of the conditions of each cell type. (IV) Cell features are extracted on Marker III and modification of the cell-type conditions. (V) The user configures the output analysis. (VI) Cell-TypeAnalyzer is run in batchmode to large image sets.

A high-level overview of the Cell-TypeAnalyzer procedure involved is shown (see Figure 2). The processing actions consist of six major stages:

- Step I: After loading the raw RGB images, we need to establish the correspondence between the RGB channels and the marker names and roles. At this point, we may perform a spatial calibration (give the pixel size in physical units) to get measurements in real length units or pixels otherwise. We may also restrict the analysis to a region of interest which must be a closed shape. The plugin shows a histogram of the pixel values in each one of the RGB channels as visual feedback.
- Step II: The next step is the identification of the cells based on Marker I. To isolate cells from their background, we offer multiple possibilities. All of them respond to an auto-thresholding with different methods^(^^20^ ^)^ to binarize the image, then a watershed transformation^(^^21^ ^)^ may be applied to separate connected cells. Next, single-cell contours are detected and boundaries traced. Once done, features are extracted from each cell, and each cell obtains a unique ID number. The plugin shows at this point a summary of the detected features through some descriptive statistics (mean, median, variance, standard deviation, minimum, maximum, quantiles, inter-quantile range, etc.). The user may now apply filters based on these features to keep only the relevant cells for their study.
- Step III: Morphological operators^(^^22^ ^)^ (erosion or dilation) may be applied to the cell contours to alter their original size. These operations allow the measurements on Marker II to be performed in a region that coincides with the area detected by Marker I (no operation), a smaller region (erosion), or a larger region (dilation). We may also perform a “Foci per nucleus”^(^^23^ ^)^ analysis to count small bright dots within each cell. Then, we will compute different features of each cell from Marker II in the selected regions. These types of features are shape descriptors (to describe cell boundaries), shape metrics, and intensity-based statistics (calculated from intensity values in each channel on each cell). Finally, we may create cell types and, to each one, add as many constraints based on the Marker II features as needed.
- Step IV: We repeat the same actions as in Step III, but now on Marker III. Then, we can add the constraints on Marker III to the definition of each cell type. Cells are assigned to each one of the types if they meet all the conditions on Markers II and III. Note that cell types can also involve conditions solely on Marker II or Marker III.
- Step V: The last interactive step allows us to configure a dynamic scatter plot to display any cell feature as a function of any other. Data points will represent relevant cells (those passing the criteria of a valid cell according to Marker I) being colored depending on their cell type or in gray if they do not fulfill the criteria of any defined cell type. Finally, we may save an XML configuration file that will allow us to run this analysis in batch mode for many images (Step VI).
- Step VI: In this step, we apply the image analysis steps defined above (cell segmentation, region operations, etc.) and classify the detected cells into the user-defined cell types to a large number of images that have been acquired with similar characteristics as the one that served to set up the analysis. This execution is performed in batch mode and produces text or Excel files with the results for each image and a summary for the whole set.

In the following paragraphs, we go over each step in more detail.

### 2.2. Step I: Marker-channel matching, spatial calibration, and sub-ROI trace

**Figure 3.**
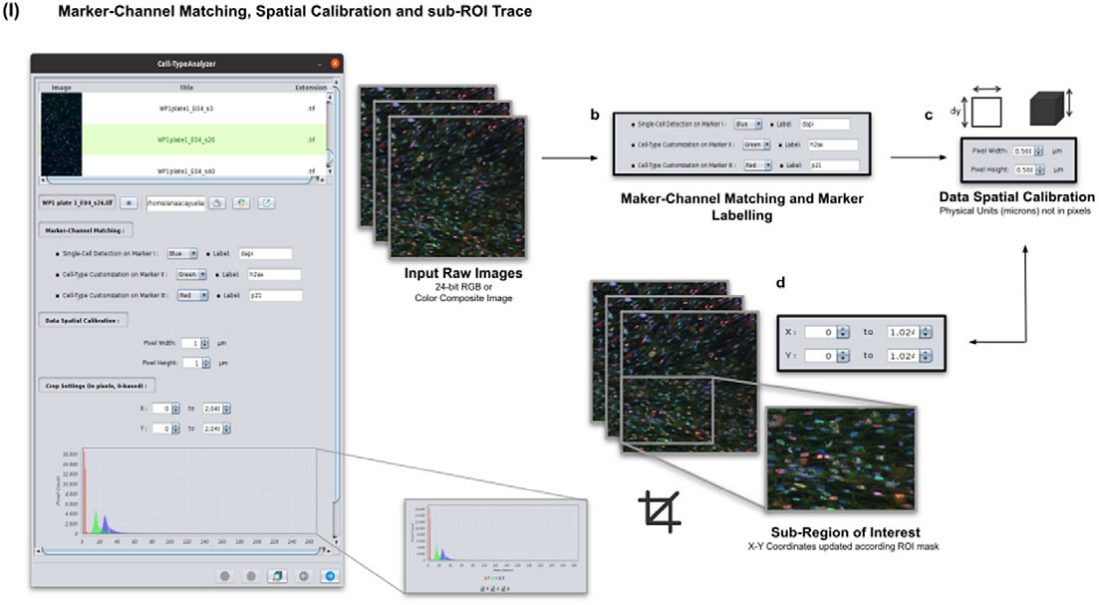
Details of Step I. Marker-channel matching, spatial calibration, and sub-ROI trace workflow. (a) Images to be processed must be in 2D single-plane RGB form: 24-bit RGB or Color Composite. (b) Via “Marker-Channel Matching,” the user must determine the matching between the RGB channels and the Markers I–III. (c) Through the “Data Spatial Calibration” panel, the user obtains all cell metrics calibrated on physical units (not in pixels) by typing the image pixel size. (d) The “Crop Settings” panel enables to draw a region of interest to be considered for analysis. All coordinates calculated throughout the plugin are updated according to the location of the closed shape. In addition, the user may inspect, by clicking on a dynamic histogram, the distribution of pixel intensities on each marker.

Color images can be loaded, and a *z*-stack is automatically generated with each separated channel and the non-split RGB image. Thanks to the instant visualization, the user has efficient control over each operation performed. Through the “Channel Settings” panel, the user can establish the marker-channel matching defining those channels used for cell segmentation (Marker I) and further cell classification (Markers II and III). The “Calibration Settings” panel enables the user to have each feature spatially calibrated on physical units rather than pixels. The user must access the pixel size usually accessible within the image metadata to properly fill these spatial calibration fields. The “Crop Settings” panel allows defining a region of interest to be analyzed. Alternatively, the user may manually draw a closed area using any shape available on ImageJ’s region-of-interest (ROI) tools^(^^24^ ^)^. If the crop option is employed, the *X*–*Y* coordinates of the detected cells are internally updated to reflect the current boundaries^(^^25^ ^)^. Finally, the last panel provides an overview of each marker’s intensity pixel value distribution with a dynamic histogram. This workflow is schematically illustrated in Figure 3.

### 2.3. Step II: Image preprocessing, cell segmentation, identification, and feature extraction on Marker I

**Figure 4.**
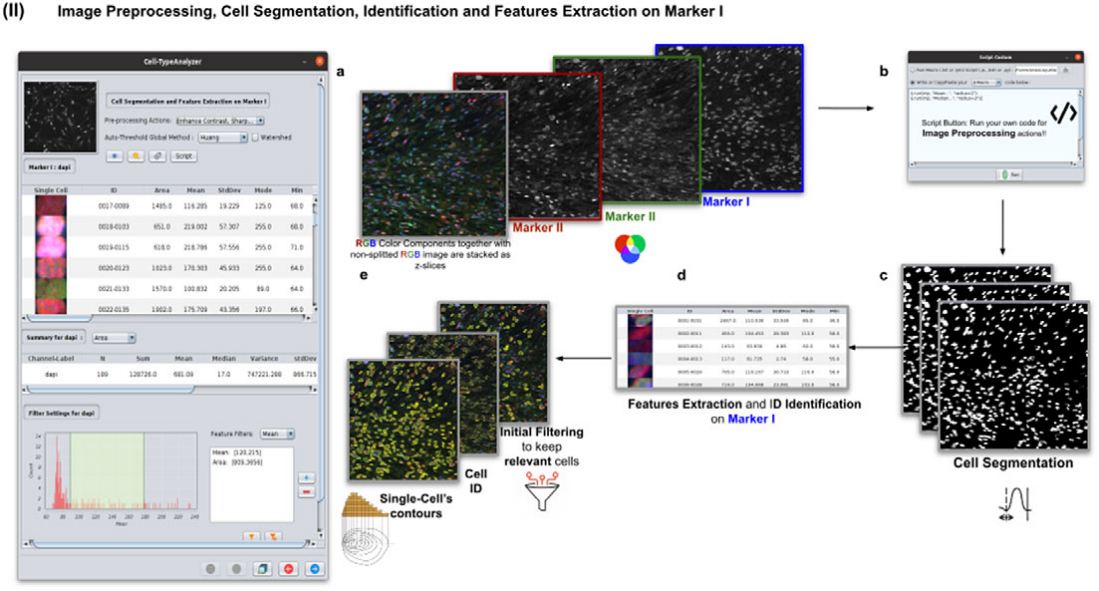
Details of Step II. Image preprocessing, cell segmentation, identification, and feature extraction on Marker I. (a) The channel corresponding to Marker I is separated from the rest of the images. (b) Now, the parameters for cell segmentation on Marker I are tuned. The user may choose from different global auto-threshold algorithms to binarize the image and isolate cells from their background. In the event of having some connected cells, watershed filtering may be applied to split touching objects. (d) Features extraction from each cell. (c) Optionally, the user may provide an image preprocessing script that facilitates the identification of the cells of interest. This is done by clicking on the “Script” button and selecting a script file or writing their own code in any of ImageJ’s supported languages. (e) Filtering to keep only relevant cells through scrolling sliders.

In this step, the user tunes the segmentation procedure to identify cells in the Marker I channel. In cases where the cell density is quite low or requires coping with a very noisy background, the user may apply some extra preprocessing actions to reduce noise using the preprocessing operations (image enhancement, correction, filtering, and de-noising) integrated by default in Cell-TypeAnalyzer. The summary of preprocessing methods is provided in Table 1. The user may apply as many preprocessing actions as needed by clicking on the Script button. A dialog window will pop up in which the user will be prompted by a script editor to write their own code in any of ImageJ’s Macro supported language without saving or even, likewise, copying it to the clipboard and pasting it on the script editor area, then run it (Figure 4). Irrespective of using those preprocessing operations integrated by default or by scripting, these are applied to the image of Marker I, prior to cell segmentation.

**Table 1.** Table listing the preprocessing operations which may be applied by default using Cell-TypeAnalyzer previous to image thresholding.

Summary of preprocessing actions		
Action	Type of action	Description
Smooth	Filter	Blurs the image replacing each pixel with the average of its 3 × 3 neighborhood.
Sharpen	Filter	Increases contrast and accentuates detail in the image replacing each pixel with a weighted average of the 3 × 3 neighborhood.
Enhance contrast	Contrast adjuster	Enhances image contrast by using either histogram stretching or histogram equalization.
Gaussian blur	Filter	Uses convolution with a Gaussian function for smoothing.
Median	Filter	Reduces image noise by replacing each pixel with the median of the neighboring pixel values.
Mean	Filter	Smooths image by replacing each pixel with the neighborhood mean.
Unsharp mask	Filter	Subtracts a blurred image copy and rescales the image to obtain the same contrast of large structures as in the input image.
Minimum	Filter	Grayscale erosion replacing each pixel in the image with the smallest pixel value in that pixel’s neighborhood.
Maximum	Filter	Grayscale dilation replacing each pixel in the image with the largest pixel value in that pixel’s neighborhood.
Variance	Filter	Highlights image edges by replacing each pixel with the neighborhood variance.

The next step is the identification of the cells of interest in the Marker I channel. More than 10 global auto-thresholding algorithms (Default, Huang, Intermodes, IsoData, Li, MaxEntropy, Mean, MinError (I), Minimum, Moments, Otsu, Percentile, RenyiEntropy, Shanbhag, Triangle, and Yen) are available to binarize the image. The user should choose the one that best suits the specificities of the images being analyzed. It is common to find cells in close contact with other cells. Binarization algorithms cannot separate them into distinct entities. For this task, we provide a watershed segmentation^(^^21^ ^)^ that works considering the output of the previous binarization. Finally, the cell contours are calculated, their boundaries traced, and features extracted (shape descriptors and intensity-based statistics) for each one of the cells are computed. To extract features from each cell, cell descriptors measure cell contours in the resulting binary image. These more than 20 different cell features computed are summarized in Tables 2 and 3.

**Table 2.** Table reporting the types of features (shape descriptors and intensity-based statistics) computed for each cell along with description using Cell-TypeAnalyzer.

Cell features summary		
Feature	Type of feature	Description
ID	ID number	Identification number of each cell contour
Area	Shape metric	Area of cell contour in squared pixels or physical units (if calibration is done)
Mean gray value (Mean)	Intensity-based statistics	Average gray value of all pixels within the cell contour
Standard deviation (StdDev)	Intensity-based statistics	Standard deviation of the gray values used to generate the mean gray value
Modal gray value (Mode)	Intensity-based statistics	Most frequent gray value within each cell contour
Min and max gray levels (Min & Max)	Intensity-based statistics	Minimum and maximum gray values within cell contour
X–Y centroid (X & Y)	Shape metric	Mean of the *x* and *y* coordinates of all pixels within each cell contour
X–Y Center of mass (XM & YM)	Shape metric	Brightness-weighted mean of the *x* and *y* coordinates of all pixels within cell contour
Perimeter (Perim.)	Shape metric	Length of each cell-contour boundary
Bounding rectangle (BX & BY, Width & Height)	Shape metric	Smallest rectangle enclosing each cell contour defined by “BX” and “BY” (the coordinates of upper left corner), and “Width” and “Height”

**Table 3.** Continuation of table reporting the types of features (shape descriptors and intensity-based statistics) computed for each cell along with description using Cell-TypeAnalyzer.

Cell features summary (continued)		
Feature	Type of feature	Description
Fit ellipse (Major, Minor, Angle)	Shape metric	Fit an ellipse to the cell contour being “Major”, “Minor” the primary and secondary axis, and “Angle” the angle among primary axis and line parallel to the *x*-axis of cell contour
Circularity (Circ.)	Shape descriptor	Being a value of 1.0 a perfect circle and a value of 0.0 an elongated shape
Aspect ratio (AR)	Shape descriptor	“Major” divided by “Minor” axis
Roundness (Round)	Shape descriptor	Inverse of “Aspect ratio”
Solidity	Shape descriptor	“Area” divided by convex Area
Feret’s diameter (Feret, FeretAngle, MinFeret, FeretX, and FeretY)	Shape metric	“FerretAngle” angle among Ferret’s diameter and parallel line to the cell contour’s *x*-axis; “MinFerret” is the minimum caliper diameter; “FerretX” and “FerretY” the starting coordinates of Ferret’s diameter
Integrated density (IntDen, RawIntDen)	Intensity-based statistics	Product of “Area” and “Mean Gray Value”
Median	Intensity-based statistics	Median value of pixels within each cell contour
Skewness (Skew)	Shape metric	Third-order moment about the mean
Kurtosis (Kurt)	Shape metric	Fourth-order moment about the mean
Area fraction (%Area)	Intensity-based statistics	Percentage of nonzero pixels

To keep the relevant cells solely, the user may filter out irrelevant cells by defining thresholds in any calculated feature. These thresholds may be chosen with the help of scrolling sliders to define the minimum and maximum values of any feature to be considered a relevant cell. The cell-type classification of the subsequent steps is only applied to those cells that have been flagged as relevant in this cell identification step based on Marker I.

### 2.4. Step III: Features extraction and customization of cell types on Marker II

**Figure 5.**
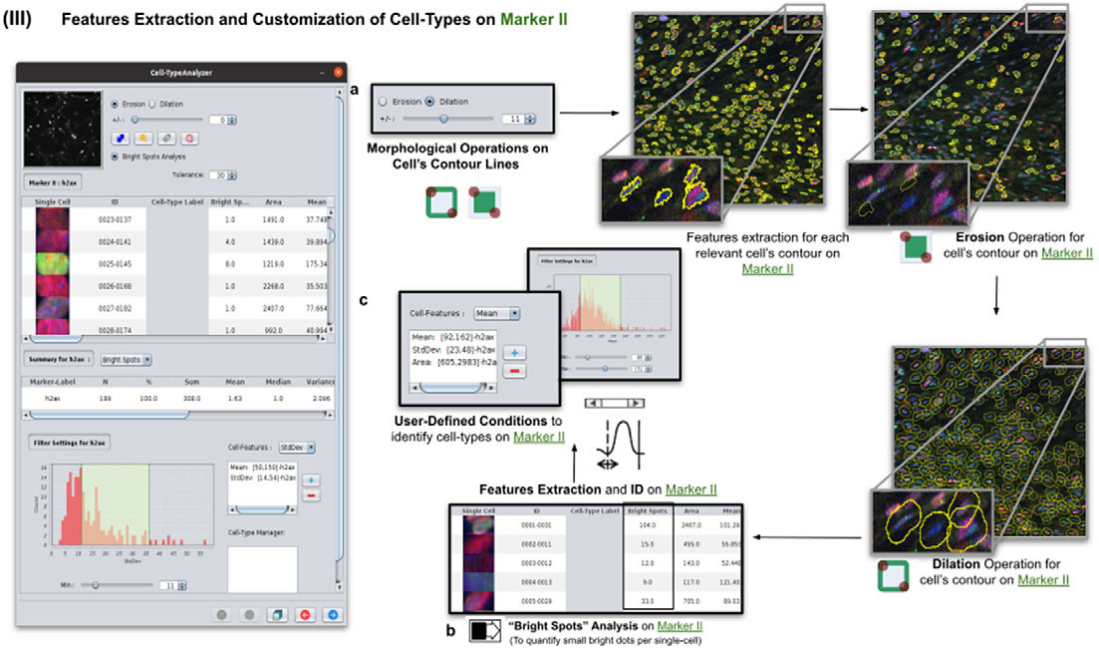
Details of Step III. Features extraction and customization of cell types on Marker II. (a) Considering as reference the relevant cells, the user may apply morphological operations (erosion and dilation) on cell contours to resize them. (b) A “Foci per nucleus” analysis^(^^23^ ^)^ may be performed whose goal is to quantify the small bright dots within each cell contour. Finally, the features extraction of relevant cells is done on Marker II, attaching this vector to the description of each relevant cell. (c) The user defines cell types based on values of the features calculated on Marker II.

Once cells have been successfully segmented on Marker I, the features extraction of relevant cells on Marker II is performed. This stage may involve morphological operations^(^^22^ ^)^ such as erosion or dilation (see Figure 5a) or, conversely, none to maintain the original cell area. If required, the user can perform a “Foci per nucleus” analysis^(^^23^ ^)^ to count all small bright dots (local maxima of pixel intensity)^(^^20^ ^)^ within each cell (see Figure 5b). This analysis has some tunable parameters like the “Tolerance” (by default 30), which acts as a local threshold (a maximum is removed from the list if it is close to another one within a distance smaller than “Tolerance”).

Features of Marker II are finally calculated for the relevant cells. This information is attached to the information already known for each cell after the analysis of Marker I. As for Marker I, this information is displayed in a data table, and a statistical summary is shown. At this point, the user may define cell types creating as many constraints as needed for the features computed on Marker II (e.g., a cell is of Type 1 if its area is between this and this value, its circularity between this and this, etc.).

### 2.5. Step IV: Features extraction and customization of cell types on Marker III

**Figure 6.**
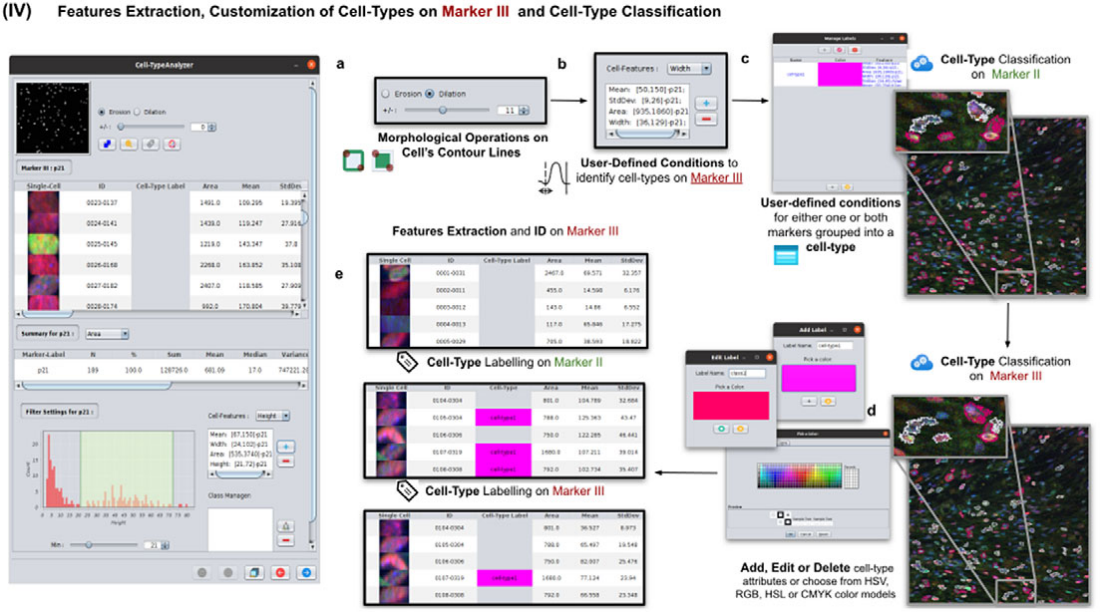
Details of Step IV. Features extraction and customization of cell types on Marker III. (a) User may either erode or dilate cell-contour lines. Then, features are extracted from relevant cells on Marker III, generating, once more, a vector for each cell. (b) Once done, the user may define conditions on any of the Marker III features to refine the definition of cell types further. (c) Cell-type labeling and coloring can be defined by the user. (d) Cell-type conditions can be iteratively defined between Steps III and IV until the desired labeling is achieved. (e) For each detected cell, a label is attached depending on which conditions it fulfills. This operation helps to refine the definition of the cell types.

This step is totally analogous to the previous one on Marker II. The definition of cell types can include conditions on any feature of both Markers II and III. A cell is classified in these types if it fulfills all the conditions of that cell type. Cells that do not fulfill any defined cell types are classified as unknown (see Figure 6).

### 2.6. Step V: Configurable outputs, dynamic marker-feature scatter plot, and cell-type metrics

**Figure 7.**
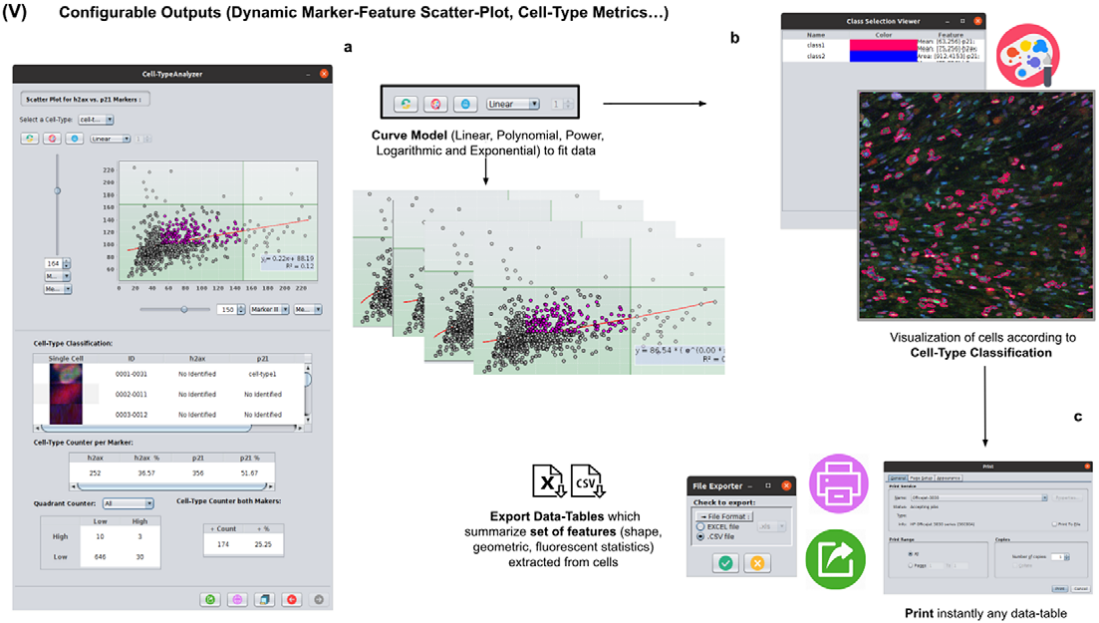
Details of Step V. Configure outputs (dynamic marker-feature scatter plot and cell-type metrics). (a) The user can dynamically plot any cell feature from either marker (Marker I, II, or III) as a function of any other. Different curve models (linear, power, polynomial, and logarithmic) can fit the data. A point represents each cell. If the cell is classified under a specific cell type, the corresponding cell-type color is used. Otherwise, cells that do not belong to any cell type are colored in gray. (b) Contours from cells belonging to a specific cell type may be visualized as outlines. (c,d) There are multiple ways of exporting the analysis, including CSV, Excel files, and PDF prints.

At this stage, the user can dynamically configure a 2D analysis (see Figure 7) to plot any cell feature extracted from the selected marker (Marker I, II, or III) as a function of any other. This functionality may be beneficial to observe relationships among features from markers. Users may choose to apply different curve-fitting models (Linear, Polynomial, Power, Logarithmic, or Exponential) to find the one that best fits data (see Figure 7c). The 2D analysis may be restricted to specific *z*-slices. This is useful to identify possible dependencies on the cell’s height within the tissue in confocal microscopy (e.g., apical vs. luminal cells). Each point represents a relevant cell. Its label determines its color on the plot. This helps to recognize cell types’ distribution patterns according to their location in specific feature planes (see Figure 7a). Cells not belonging to any cell type are colored in gray. As an additional way to explore the set of cells, the user may define thresholds for each plotted feature (see Figure 7a). These thresholds divide the feature space into four quadrants, and the number of cells in each quadrant is counted and displayed as a table.

If the user is satisfied with the analysis performed on several input images, the whole analysis description can be saved as an XML file used by the plugin in batch mode (see the next section).

### 2.7. Step VI: Running Cell-TypeAnalyzer in batch mode

**Figure 8.**
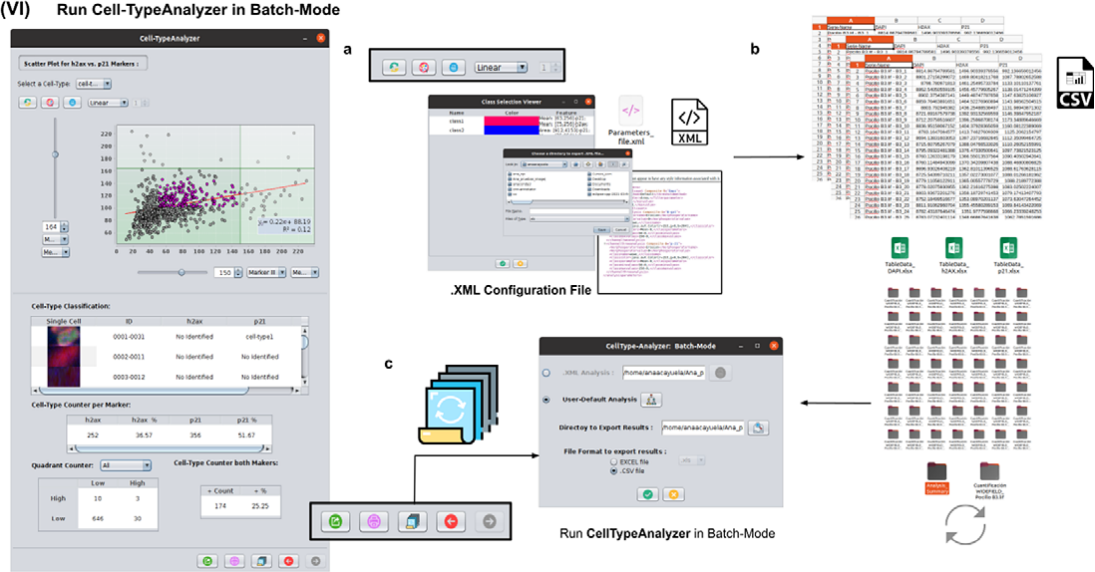
Details of Step VI. Execution of Cell-TypeAnalyzer in batch mode. (a) The user may save an XML configuration file that summarizes all the steps required, and it will be used to run Cell-TypeAnalyzer for large sets of images. (b) Examples of output files generated. (c) Graphical user interface for batch mode.

To achieve the most accurate batch-mode analysis (see Figure 8), it is advised to perform prior tests to find the most suitable parameters on a subset of images before applying it to a large batch. The batch-mode GUI will ask the user to load the XML configuration file containing the whole list of user-defined processing actions. This file is saved in Step V. Using XML is advantageous because it is editable. Thus, the user can easily change the analysis without reopening the GUI and designing the analysis from scratch. Then, the user must supply the directory with the input images and the directory for the outputs.

## 3. Experimental Validation

To validate Cell-TypeAnalyzer and demonstrate how versatile it is in solving specific biological problems in several image sets, we propose different applications in which this tool may be customized.

### 3.1. Comparing cell-quantification using confocal and widefield microscopy

**Figure 9.**
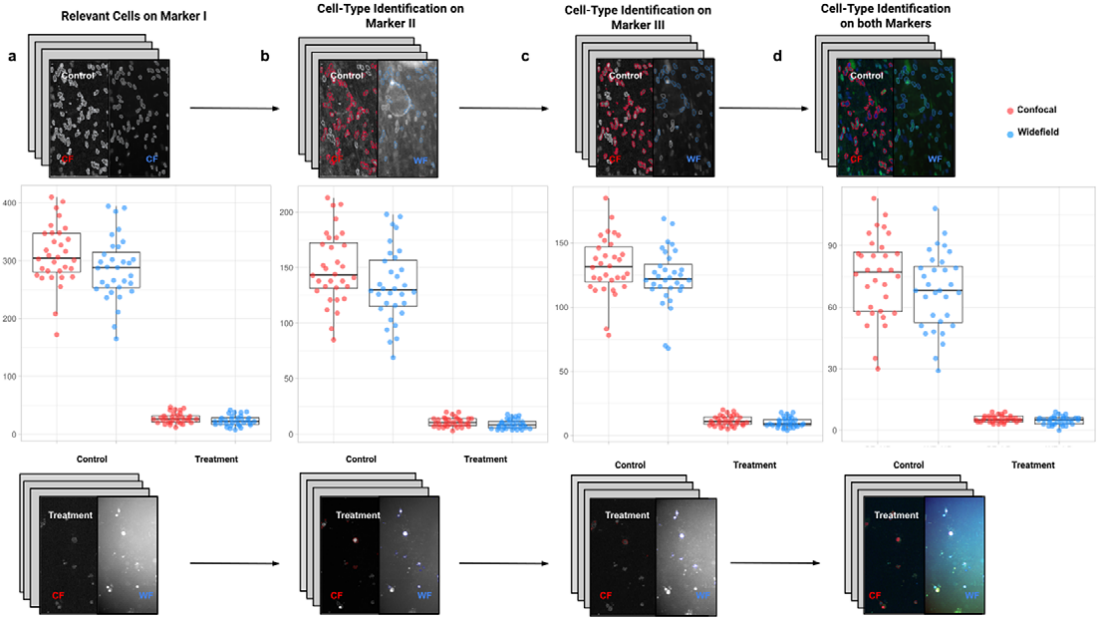
Box-whisker plots summarizing the distribution of both control and treatment groups from confocal and widefield microscopes values. Each point will be representing the total number of cells quantified for each analyzed well. (a) Data points calculated by quantifying relevant detections for control and treatment samples on Marker I (DAPI). The distribution charts reveal nonsignificant differences between microscopes. (b,c) Data points calculated by quantifying cells identified within a specific cell type on Markers II and II, respectively. The distribution reveals nonsignificant differences among microscope tested. (d) Data points calculated by quantifying cells that are identified simultaneously as a specific cell type for both markers.

In the first application, we compared the count of cells of a given type using Confocal or Widefield microscopy^(^^26^ ^)^. We did not expect a significant difference between the two kinds of microscopy despite their different appearances in this experiment. We had two different cell preparations: control and treated cells (see Figure 9). A larger growth rate characterized the control group, and microscopy fields showed a higher cell density, while the treated group had a lower cell density^(^^27^ ^)^. Images in each well were acquired containing channels for 4',6-Diamidino-2-Phenylindole (double stranded DNA staining) (DAPI) as Marker I; this marker is a dye for targeting the cell nuclei^(^^28^ ^)^. As Marker II, we used Rabbit antihuman p21 (at 1:500 dilution), and antibody labeled cells were visualized with Goat anti-rabbit secondary antibody directly conjugated to fluorochrome Alexa 647 (at 1:500 dilution). As Marker III, we used Mouse antihuman phospho-histone H2AX (at 1:500 dilution), a biomarker to recognize DNA damage^(^^29^ ^)^, being visualized with Goat anti-mouse secondary antibody directly conjugated to fluorochrome Alexa 488. Both Confocal multispectral system Leica STELLARIS 5 system and Leica DMi8 S Widefield epifluorescence were employed for the image acquisition. Each field of view was operated at a map resolution format of 1,024 × 1,024 pixels with each channel at 16-bit intensity resolution. The confocal images were acquired with an HC PL APO CS2 20 × /0.75 DRY objective and have a pixel size of 0.758 × 0.758 microns. The widefield images were acquired with an HC PL FLUOTAR L 20 × /0.40 DRY objective and have a pixel size of 0.65 × 0.65 microns. Images were acquired with a step size of 2.5 microns and intervals of approximately 9 s per image (44 sites per well), resulting in barely 10 min per well. A total of 128 wells were imaged using three channels, which resulted in 384 grayscale images. A dataset of 32 images per group (confocal-control, confocal-treated, widefield-control, and widefield-treated) was collected.

For the image analysis, we used Otsu’s binarization to identify cell nuclei in Marker I. We did not need to use watershed segmentation to separate nearby cells. We removed all cells whose nucleus had a Marker I area below 5 pixels. We performed a “Foci per nucleus” analysis on Marker II. We defined a single cell type by requiring cells to have an average intensity in Markers II and III above the average intensity in Marker I and with at least eight foci. The analysis time per image was 6 s in a laptop Alienware M15 8th Gen Intel Core i7-8750H (4.1 GHz). The results of Cell-TypeAnalyzer in batch mode are shown in Figure 9. In Table 4, we compare the mean (two-sample *t*-test) between the confocal and widefield imaging. None of the tests could be rejected at a confidence level of 95%. This comparison shows that imaging with confocal or widefield microscopy does not make any statistically significant difference for this experiment. Cell-TypeAnalyzer was instrumental in automating this comparison, which would have been much more tedious if manual counting was required.

**Table 4.** Table showing descriptive statistics for both cell populations (Confocal and Widefield) depending on Control or Treatment conditions. Panel A: Control—means and distributions are nonsignificantly different between Widefield and Confocal microscopes at the 0.05 level in *t*-test. Panel B: Treatment—means and distributions are nonsignificantly different between Widefield and Confocal microscopes at the 95% confidence level in *t*-test. Since *p*-value > .05, the average of WF’s population cannot be rejected from being equal to the average of the CF’s population.

	Marker I		Marker II		Marker III		Cell types	
	WF^a^	CF^b^	WF	CF	WF	CF	WF	CF
Panel A: Control								
Mean	285.59	309.63	134.25	149.56	123.41	132.16	67.84	74.72
SD	54.17	51.9	32.99	31.95	21.88	22.62	18.64	19.88
*p*-value	0.075		0.064		0.121		0.159	
Effect size	0.45		0.47		0.39		0.36	
*r* ^c^	0.978		0.983		0.973		0.987	
Panel B: Treatment								
Mean	23.47	27.91	9.03	11	10.06	11.41	4.69	5.53
SD	8.97	9.06	4.26	4.24	3.76	4.02	2.05	1.67
*p*-value	0.053		0.069		0.172		0.076	
Effect size	0.49		0.46		0.35		0.45	
*r* ^c^	0.989		0.933		0.885		0.814	

*Notes:* Statistical significance depending on *p*-value at the *p* < .05 level. *p*-values were determined by using two-sample *t*-test for expected difference between two populations’ mean (*n* = *32*).

a Widefield microscope technique.

b Confocal microscope technique.

c Pearson correlation coefficient.

### 3.2. Evaluating morphology of known phenotypes in HeLa cells

This second application proposes a general approach to identify and get subsequent phenotype classification of single cells within cell populations based on morphological changes.

This study used the Cell-TypeAnalyzer plugin in batch mode to efficiently extract shape descriptors and features from cells to analyze their morphology and obtain fluorescence statistics from nuclear and cytoskeletal markers. Each cell was described by a vector of more than 30 descriptors measured for each fluorescent marker (DNA, Tubulin, and Actin).

**Figure 10.**
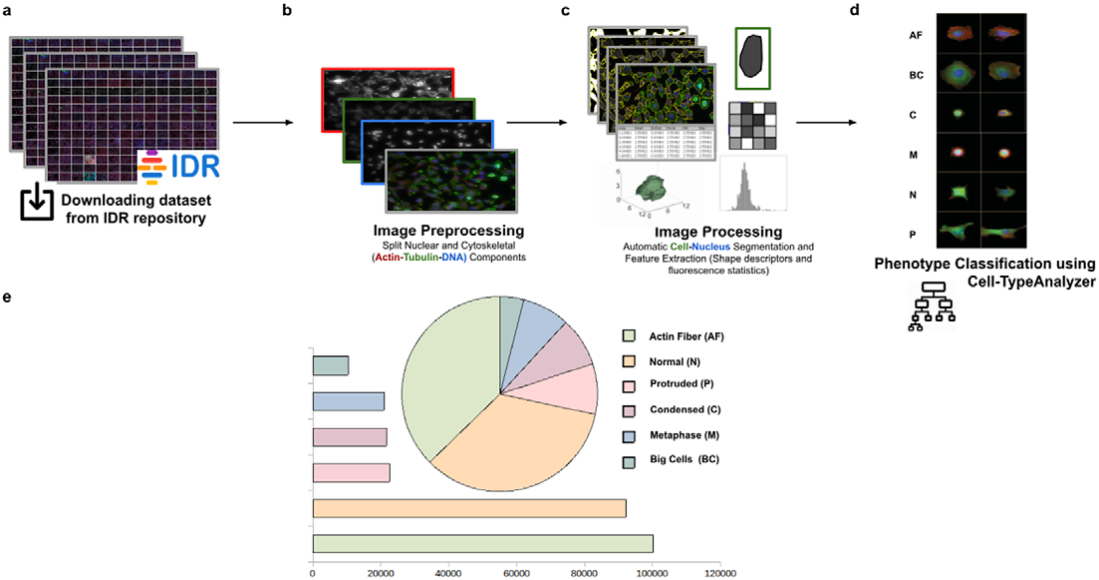
Scheme of semiautomated analysis of raw images for classifying cellular phenotypes in HeLa cells. (a) The full dataset of HeLa cells images to be analyzed was downloaded from the image data resource repository. (b) Image preprocessing actions to get the separated nuclear (DNA) and cytoskeletal (Actin and Tubulin) components were applied. (c) Image processing actions for Cell–Nucleus segmentation and subsequent identification. A vector describes each cell based on shape descriptors, geometry, and fluorescence statistics. (d) Cells were classified into Actin Fiber (AF), Big cells (BC), Condensed (C), Metaphase (M), Normal (N), and Protruded (P) cell-type classes depending on user-defined feature conditions set for each case. (e) Quantification results of classifying HeLa cells belonging to each cellular phenotype.

Using this tool, we established a semiautomated method for identifying distinct phenotypes from subsequent classification, according to classes that are user-defined for each cell type. These classes consist of parameters based on morphology and cell area to describe their protrusion or elongation. HeLa cells in this application were first detected, identified by an ID number, and finally, classified into different cell types: Actin fiber (AF), Big cells (BC), Condensed cells (C), Metaphase cells (M), Normal cells (N), and Protruded cells (P). The full dataset of HeLa cells was downloaded from the image data resource (IDR)^(^^30^ ^)^, a public repository of high-quality bio-image datasets from published scientific studies. Specifically, images were selected from the “idr0012-fuchs-cellmorph” dataset^(^^31^ ^)^. This dataset consists of 22,839 siRNA-mediated knockdowns on HeLa cells in which genes were clustered, and their function predicted on a genome-wide scale. The workflow for cell-type classification (see Figure 10) starts with the “Splitting multi-channel images” command, which is called automatically by Cell-TypeAnalyzer. This command is used for color image processing since it splits the RGB images into their respective nuclear (DNA) and cytoskeletal (Actin and Tubulin) components. Nuclei and cytoplasm boundaries were isolated through segmentation using the “Auto-Threshold” Otsu’s and Huang’s methods, respectively. The watershed segmentation was applied to separate touching nuclear or cytoskeletal structures. Once these regions were isolated, features were measured from both cytoskeletal fluorescent markers (Tubulin and Actin) for each cell. An initial filtering was applied to remove those regions having an area in pixels smaller than 20. The remaining cells were classified depending on their quantified fluorescent intensity on the Actin marker and their respective circularity values. Those cells having more intensity in the Tubulin marker than the Actin marker and a circularity value located in the Q4 quartile of the distribution for circularity were classified as Metaphase (M) cells. Otherwise, cells were classified as Actin fibers (AF) class. Cells were classified into the Big cells (BC) class if their area belonged to the Q4 quartile. The remaining cells were considered as candidates to belong to the Normal (N), Condensed (C), or Protruded (P) cell type. This classification was performed as a function of the circularity: Protruded (if the circularity was in the Q1 quartile), Normal (Q2 or Q3), and Condensed (Q4). The proportions of cells in each one of the types are similar to the one originally reported in Reference (31).

### 3.3. Classifying morphology in Spirochaete bacteria on dark-field microscopy

This third application proposes a widely applicable analysis workflow to detect, identify by ID, quantify, and get subsequent morphological phenotyping of *Spirochaete* bacteria in blood. We applied our tool on all dataset images in Reference (32), a total of 366 dark-field microscopy images. It must be noted that these images are monochromatic, showing that our tool is not restricted to the analysis of multichannel images. The phenotype classification was done using the Cell-TypeAnalyzer plugin in batch mode. Cell features (shape descriptors and intensity-based statistics) were automatically extracted from every cell as a vector used to classify them into different morphological classes.

The evaluation of bacterial single-cell contours may be instrumental in getting new insights into the morphological changes due to a wide range of processes that external perturbations may induce and thus reflected in the cell shape. Although *Spirochaete* organisms normally show stable, well-defined shapes, these single-cell microbes may change their morphology in response to certain environmental signals or even, depending on their life-cycle stage. The following morphological cell types were defined: Blood Cells (BC), Normal (N), Small (S), Elongated (E), and Round (R). In this analysis, around 53,000 cells were automatically segmented, identified, and classified into each cell-type class.

**Figure 11.**
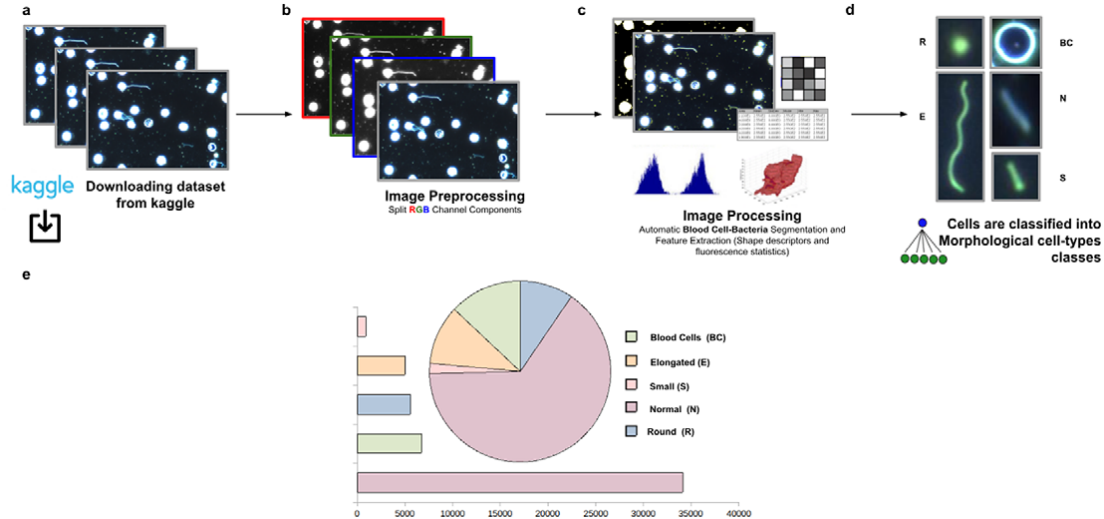
Scheme of semiautomated analysis of raw images for classifying *Spirochaete* bacteria in the blood. (a) The full dataset of images to be analyzed was downloaded from Kaggle. (b) Image preprocessing actions to get the separated channel components were applied. (c) Image Processing actions for Blood Cells–Bacteria segmentation and subsequent identification. A vector describes each cell based on shape descriptors, geometry, and fluorescence statistics. (d) Cells were classified into Blood Cells (BC), Round (R), Elongated (E), Small (S), and Normal (N) cell-type classes. (e) Quantification results of classifying cells belonging to each morphological class.

The workflow for phenotype classification (see Figure 11) on bacterial cells starts with the “Splitting multi-channel images” command to separate the RGB images to their respective color components. Blood cells and *Spirochaete* bacteria boundaries were isolated through segmentation using the “Auto-Threshold” Otsu’s method to binarize the image. Then, the watershed segmentation was applied. The “Fill Holes” command was called obtaining more homogeneous regions. Once these regions of interest were isolated, cell features described in Section 5 were calculated for every cell. Candidate cells were first classified as blood cells or bacteria cells depending on their area in pixels. Subsequently, bacteria cells with circularity values located in the Q4 quartile were classified as Round (R) cells. The remaining cells were classified as Elongated (E), Small (S), or Normal (N) according to their area. Hence, bacteria cells showing an area in pixels located in the Q4 quartile were labeled as Elongated €, then those having area values belonging to Q1 were identified as Small (S), and finally, those located both at inter-quartile range were classified as Normal (N).

## 4. Discussion

Cell-TypeAnalyzer was developed to facilitate researchers the single-cell identification and subsequent cell-type classification under user-defined conditions. Furthermore, Cell-TypeAnalyzer may measure large sets of images with many cell types of interest previously defined by the user in a large variety of biological samples, an aspect which is increasingly recognized as crucial to improve our understanding of how genetic and environmental factors give rise to changes in organisms or even in their behavior^(^^33^ ^)^.

Two of the most popular image analysis software for identifying and quantifying cell phenotype are CellProfiler^(^^34^ ^)^ and CellProfiler Analyst (CPA)^(^^35^ ^)^. CellProfiler is a flexible and open-source image analysis software package which allows users to mix and match modules to create their own customized image analysis pipelines without extensive programming skills. CPA was released in 2008 and marked a great progress in fully automated phenotypic analysis including modern statistical learning methods to identify specific cell phenotypes. This software (directly interfaced to CellProfiler) enabled biologists to define a bunch of phenotypes as well as create annotations for single cells to train supervised machine learning algorithm to further predict phenotypes on unseen data. Notwithstanding the first release of CellProfiler exhibited several constraints in terms of the definition of classes (only supported two classes: positive and negative), and on its behalf, CPA provided a small number of machine learning algorithm available for classification (only GentleBoost), both tools were extensively used worldwide. Nowadays, those limitations have been amply overcome since recent releases have incorporated the definition of multiple phenotype classes as well as different machine learning algorithms are currently supported.

Having shown that trends in phenotypic analysis go through using platforms in fully automated mode, we would like to make a comparison with our tool for cell-type classification functioning in semiautomatic mode. All of these widely used tools which perform phenotypic analysis on images of cell-based assays are designed to operate fully automatically. This aspect is absolutely great in terms of processing speed and objectiveness, but sometimes might compromise accuracy regarding the user interpretation of extracted features which are prone to unexpected errors in case of users not having extensive experience. Moreover, this lack of interpretation may be more obvious as regards the complex structure of deep learning neural networks as well as the sophisticated of machine learning models that regularly require a prior knowledge of the field to be applied. On the other hand, semiautomated tools always require user input and interaction along with expert validation to extract the required information accurately and these methods, normally, are quite dependent on the quality of raw image data. Thereby such manual intervention might be certainly time-consuming, and it may introduce subjective bias leading to hind the implementation of these approaches to large sets of images.

In this context, there is a general growing need of universal tools for varying image conditions to accomplish semiautomated phenotypic analysis that enable cell-type classification within ImageJ or Fiji ecosystem. The development of Cell-TypeAnalyzer within ImageJ might help to remove this bottleneck in experimental pipelines which often involve complex workflows for a non-experienced community, offering a user-friendly solution relying less on fully automation. Moreover, Cell-TypeAnalyzer may be a worthy contribution with many options to customize cell-type analysis on multi-fluorescent microscopy images containing hundreds of objects, but it might be broadly applicable to other heterogeneous microscopy samples.

Consequently, an important difference between the more advanced software described above and our approach is that the advanced tools do not allow the user to fine-tune the parameters used for the classification in an understandable way, making outputs challenging to verify by user. For that reason, we have designed Cell-TypeAnalyzer, that is modularly designed through a simple wizard-like GUI to visually guide user to each step of the analysis offering an instant visualization of the outputs for each marker, hence enabling manual verification across the navigation back and forward through wizards. As is already the case of CPA, which has an interactive GUI to view images, Cell-TypeAnalyzer allows user to manually scroll through a gallery view of image thumbnails corresponding to the input folder in which samples are located to directly being chosen by user for being analyzed avoiding the tedious of browsing directories. Additionally, Cell-TypeAnalyzer benefits from ImageJ ecosystem, which is probably the best-known, flexible, and longest-lived software for biomedical sciences and beyond. In consequence, Cell-TypeAnalyzer leverages from a lot of plugins for scientific image processing included within its distribution, such as Bio-Formats library, which deals with more than 150 different file formats. Even though CellProfiler offers quite powerful tools for detecting, quantifying, and describing cell morphology, Cell-TypeAnalyzer benefits from a large library of tools within Fiji distribution such as MorphoLibJ^(^^22^ ^)^ for morphological filtering as well as reconstruction and global ImageJ thresholding for binarization/segmentation. Additionally, more experienced users may develop their own macro programs to automate image preprocessing actions such as brightness correction, pixel and geometric transformations, or even image filtering and restoration using ImageJ macros action integrated within Cell-TypeAnalyzer plugin. At this point, it must be noted that Cell-TypeAnalyzer was not developed to remove noise or enhance quality from images, although it provides preprocessing tools to improve cell detection and cell-type characterization. Best practices for acquisition are, whenever possible, recommended before using this tool. As is generally known, ImageJ was traditionally designed for single-image processing. On the contrary, CellProfiler was originally devised for building large-scale and modular analysis pipelines^(^^36^ ^)^. In this sense, Cell-TypeAnalyzer introduces the batch processing to implement the cell-type analysis based on user-defined conditions on large image datasets (once the user is satisfied with single-image results), avoiding both the individual and tedious processing of each image. Regarding data visualization, such as with CPA software which offers heatmaps, boxplots, and histograms, Cell-TypeAnalyzer allows users for data visualization and exploration to easily drill down the cell-type classification results using dynamic scatter plots.

Regarding usability, the semiautomated analysis provided by Cell-TypeAnalyzer does not require any programming proficiency thanks to its user-friendly wizard-like GUI and its quite intuitive visualization settings. Nonetheless, some instructions and video tutorials are supplied in our documentation (https://github.com/QuantitativeImageAnalysisUnitCNB/CellTypeAnalyzer). As already happens with CPA software, Cell-TypeAnalyzer code is entirely open-source, and it does not require any commercial license. In terms of functionality, as in the case of CPA software, time-lapse data are not supported for analysis being solely possible to be used with static images; instead, Cell-TypeAnalyzer is able to process each slice from time-lapse image independently. CPA relies on CellProfiler to extract a huge amount of features to describe each cell presuming that user has prior knowledge of cell types present on images thus whether there is a large set of images, it is impossible to identify all the significant cell types by visual inspection. For these reasons, Cell-TypeAnalyzer computes features of each cell readily understandable for average users such us shape descriptors (perimeter, area, and roundness) or intensity-based statistics in each channel within each segmented cell compartment.

On the other hand, these days, biologists are increasingly becoming more qualified users, which is leading to a deeper understanding of the data. As opposed to machine learning approaches in which the user is requested to costly label many input cells to train the underlying classifier, Cell-TypeAnalyzer suggests cell-type classification based on simple rules on the calculated features. A further consideration of Cell-TypeAnalyzer related to CPA is the potential limitation of being restricted to maximum of three input image channels for cell-type analysis.

**Figure 12.**
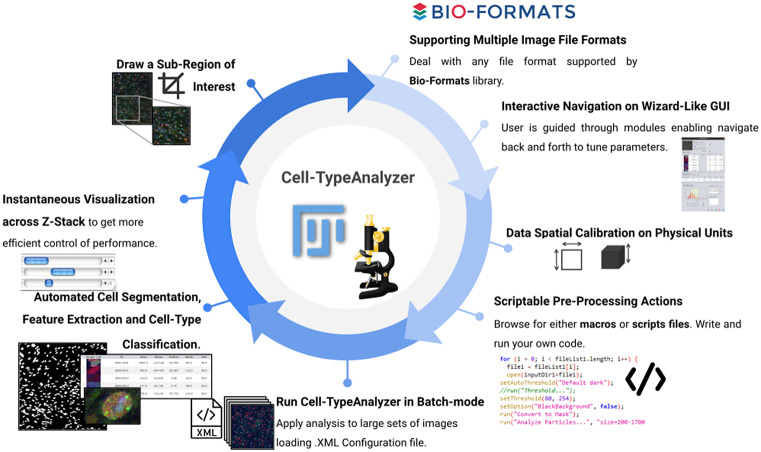
Schematic description of Cell-TypeAnalyzer main functionalities. It is an open-source Fiji or ImageJ plugin for the semiautomated classification of cells according to specific cell types defined by the user. It offers a flexible and modular solution for users through an intuitive graphical user interface. It can deal with multiple image formats supported by the Bio-Formats library. It is also easily scriptable to perform preprocessing actions before cell segmentation and feature extraction. Cell-TypeAnalyzer allows the user to calibrate metrics on physical units, not in pixels, together with having instant visualization of each step of the analysis.

Overall, by leveraging a blend of semiautomatic and manual tools, Cell-TypeAnalyzer may achieve an accurate and efficient single-cell detection in images of not well-separated objects as well as high speed in batch processing, allowing identification of hundreds of cell types per minute. By contrast, in cases of images where objects are touching, segmentation may be a tough task; therefore, manual verification along with the adjustment of preprocessing actions is required. Finally, the batch processing will generate a folder for each processed image along with a summary folder per directory processed, which makes it possible to researchers easily examine outputs to determine how different are the identified cell types. The data tables will be saved using the common CSV file format, enabling its use in any spreadsheet application for further complex analyses, which makes the data accessible to researchers without programming experience. All together, these features make Cell-TypeAnalyzer, despite some limitations, an accessible plugin for researchers at different levels of domain which facilitates cell-type classification under user-defined conditions at different phases of the cell cycle (Figure 12).

## 5. Methods and Materials

### 5.1. Confocal and widefield microscopy

Images were acquired with a Confocal multispectral system Leica STELLARIS 5, using three laser lines: 405, 488, and 638 nm for DAPI, Alexa 488, and Alexa 647 excitation, respectively, and three Power HyD S spectral detectors for the fluorochromes’ emission detection and integrated software module for real-time multidimensional super-resolution multidimensional image detection and processing (Lightning) and Leica DMi8 S widefield epifluorescence microscope with led lines: 405, 490, and 635 nm for DAPI, Alexa 488, and Alexa 647 excitation with the appropriate filter cubes to detect the specific emission of the fluorochromes, and a Hamamatsu Flash 4 sCMOS digital camera for image detection.

### 5.2. Development and implementation

Cell-TypeAnalyzer was developed in Eclipse integrated development environment^(^^37^ ^)^ for Java Developers version 2019-12 (4.14.0), an open-source platform mainly written in Java and used in computer programming for computer programming developing user-friendly Java applications. Cell-TypeAnalyzer is a Java application that inherits from ImageJ’s plugin class, thus extending ImageJ’s ecosystem. The core software and GUI were built using Java 8. Plots and histograms were implemented using the JFreeChart library. For reading the input images, we used the Bio-Formats library^(^^19^ ^)^. For handling XML files, we used JDom, and for handling Microsoft Office Formats (.xls and .xlsx), we used Apache POI libraries.

### 5.3. Installing in Fiji or ImageJ

Cell-TypeAnalyzer runs as a plugin of Fiji or ImageJ (https://imagej.nih.gov/ij/download.html) and consequently can be executed in Windows, Mac OS, or Linux systems. Cell-TypeAnalyzer plugin does not have an updated site yet. To install it, the file CellTypeAnalyzer_jar must be downloaded from https://github.com/QuantitativeImageAnalysisUnitCNB/CellTypeAnalyzer and moved into the ImageJ/Fiji plugins subfolder. Alternatively, it can be dragged and dropped into the ImageJ/Fiji main window or, optionally, installed through ImageJ/Fiji menu bar Plugins 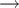 Install 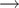 Path to File. After installing the plugin, ImageJ or Fiji must be restarted.

### 5.4. Supported image file formats

Cell-TypeAnalyzer deals with a wide range of file formats using Bio-Formats^(^^19^ ^)^, an open-source library from life sciences supporting or reading almost any image format or multidimensional data as *z*-stacks, time series, or multiplexed images, keeping metadata easily accessible. In case of loading a Leica Image File^(^^38^ ^)^ whose extension is .lif, which is a file format allowing storing several image series in the same file, our software is capable of extracting each image automatically as a single TIFF file, keeping the original pixel values and spatial calibration. On top of that, the user has access to a list of images that are available during the whole procedure for updating analysis as many times as needed. Regarding the limitation of usage, Cell-TypeAnalyzer is restricted to images in 2D single-plane RGB form: 24-bit RGB or Color Composite.

### 5.5. Code availability

Source code and documentation for the plugin are available at https://github.com/QuantitativeImageAnalysisUnitCNB/CellTypeAnalyzer.

## 6. Conclusions

This paper presents Cell-TypeAnalyzer, a plugin for automatically detecting and semiautomatically classifying cells according to very flexible cell-type definitions in multiple microscope image files. This tool was developed as a plugin working under both ImageJ and Fiji platforms. The implemented procedure consists of image preprocessing actions, cell segmentation, cell characterization through the extraction of features in RGB channels, and cell classification. This tool was designed to interactively guide users through various modules, allowing navigating back and forth to tune parameters or review processing actions while performing cell classification. Therefore, Cell-TypeAnalyzer offers a user-friendly, generic, and flexible strategy that can be applied to a wide range of biological challenges to examine relationships among cells that might reveal worthy new biological insights.

## Data Availability

Source code and documentation for the plugin are available at https://github.com/QuantitativeImageAnalysisUnitCNB/CellTypeAnalyzer.

## Supplementary material

For supplementary material accompanying this paper visit https://doi.org/10.1017/S2633903X22000058. click here to view supplementary material
